# Evaluation of 1,10-phenanthroline-based hydroxamate derivative as dual histone deacetylases/ribonucleotide reductase inhibitor with antitumor activities

**DOI:** 10.1007/s40199-024-00514-1

**Published:** 2024-04-29

**Authors:** Manasa Gangadhar Shetty, Padmini Pai, Bipasa Dey, Kapaettu Satyamoorthy, Suranjan Shil, Usha Yogendra Nayak, Ashwini T, Babitha Kampa Sundara

**Affiliations:** 1 Department of Biophysics, Manipal School of Life Sciences, Manipal Academy of Higher Education, Manipal, 576104 Karnataka India; 2 Shri Dharmasthala Manjunatheshwara (SDM) University, Manjushree Nagar, Sattur, Dharwad, 580009 Karnataka India; 3 Department of Chemistry, Manipal Centre for Natural Sciences (Centre of Excellence), Manipal Academy of Higher Education, Manipal, 576104 Karnataka India; 4 Department of Pharmaceutics, Manipal College of Pharmaceutical Sciences, Manipal Academy of Higher Education, Manipal, Karnataka 576104 India

**Keywords:** 1,10-Phenanthroline, Hydroxamic acid, HDAC, RR, Anti-cancer, Dual targeting

## Abstract

**Background:**

Aberrant expression of histone deacetylases (HDACs) and ribonucleotide reductase (RR) enzymes are commonly observed in various cancers. Researchers are focusing on these enzymes in cancer studies with the aim of developing effective chemotherapeutic drugs for cancer treatment. Targeting both HDAC and RR simultaneously with a dual HDAC/RR inhibitor has exhibited enhanced effectiveness compared to monotherapy in cancer treatment, making it a promising strategy.

**Objectives:**

The objective of the study is to synthesize and assess the anti-cancer properties of a 1,10-phenanthroline-based hydroxamate derivative, characterizing it as a novel dual HDAC/RR inhibitor.

**Methods:**

The *N*^1^-hydroxy-*N*^8^-(1,10-phenanthrolin-5-yl)octanediamide (PA), a 1,10-phenanthroline-based hydroxamate derivative, was synthesized and structurally characterized. The compound was subjected to in vitro assessments of its anti-cancer, HDAC, and RR inhibitory activities. In silico docking and molecular dynamics simulations were further studied to explore its interactions with HDACs and RRM2.

**Results:**

The structurally confirmed PA exhibited antiproliferative activity in SiHa cells with an IC_50_ of 16.43 μM. It displayed potent inhibitory activity against HDAC and RR with IC_50_ values of 10.80 μM and 9.34 μM, respectively. Co-inhibition of HDAC and RR resulted in apoptosis-induced cell death in SiHa cells, mediated by the accumulation of reactive oxygen species (ROS). In silico docking studies demonstrated that PA can effectively bind to the active sites of HDAC isoforms and RRM2. Furthermore, PA demonstrated a more favorable interaction with HDAC7, displaying a docking score of -9.633 kcal/mol, as compared to the standard HDAC inhibitor suberoylanilide hydroxamic acid (SAHA), which exhibited a docking score of -8.244 kcal/mol against HDAC7.

**Conclusion:**

The present study emphasizes the prospect of designing a potential 1,10-phenanthroline hydroxamic acid derivative as a novel dual HDAC and RR-inhibiting anti-cancer molecule.

**Graphical Abstract:**

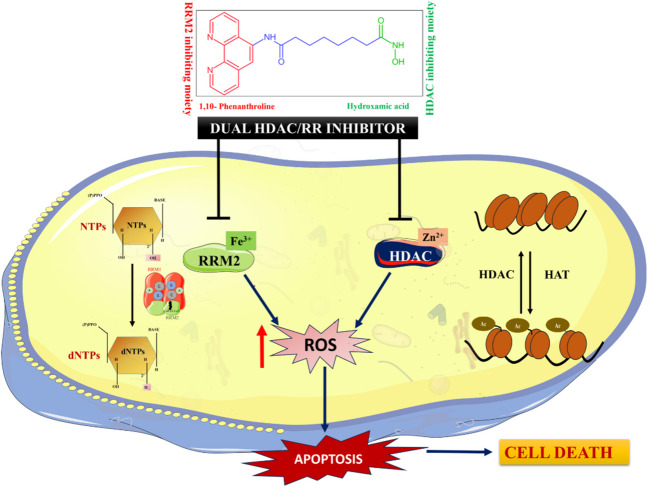

**Supplementary Information:**

The online version contains supplementary material available at 10.1007/s40199-024-00514-1.

## Introduction

Genetic abnormalities and epigenetic alterations play critical roles in various stages of carcinogenesis, including initiation, progression, invasion, and metastasis [1]. Unlike genetic abnormalities, epigenetic modifications are reversible and responsive to extrinsic factors, making them valuable targets for cancer treatments [2, 3]. Histone proteins, which scaffold chromatin and control gene expression, undergo post-translational modifications [4]. Acetylation is one of the important post-translational modifications and is regulated by two key enzymes, namely, histone acetyltransferases (HATs) and histone deacetylases (HDACs) [5]. The latter, comprising eighteen human HDAC isoforms, are promising targets for anti-cancer therapeutics, as HDAC inhibitors have demonstrated efficacy in inhibiting cancer proliferation by re-expressing suppressed regulatory genes [6, 7].

Due to the complex nature of the tumor, combination chemotherapy often yields better therapeutic outcomes due to its ability to act on multiple mechanisms of tumor growth [8]. However, it has shortcomings, including poor patient compliance, drug interactions, high cost, and increased toxicity [9, 10]. Therefore, developing dual or multi-targeting anti-cancer hybrid molecules capable of targeting multiple pathways at a favorable concentration is an attractive option. Hybrid molecules, exemplified by FDA-approved drugs like Lapatinib, Duvelisib, Dasatinib, Vandetanib, and Sorafenib, show promise in overcoming the limitations of mono-therapeutic cancer medications [11–15].

Several studies have reported significant cancer growth inhibition and apoptotic synergy when HDAC inhibitors are combined with ribonucleotide reductase (RR) inhibitors [16–21]. RR enzyme with subunits RRM1 and RRM2 is responsible for deoxynucleotide triphosphate (dNTP) synthesis [22]. It is overexpressed in chemotherapy-resistant cancer cells [23]. The smaller subunit of RR (RRM2) houses di-ferric (two ferric ions) Fe^3+^ ions and a tyrosyl radical center, making iron chelators and radical scavengers an effective classes of RR inhibitors [24].

**Fig. 1:**
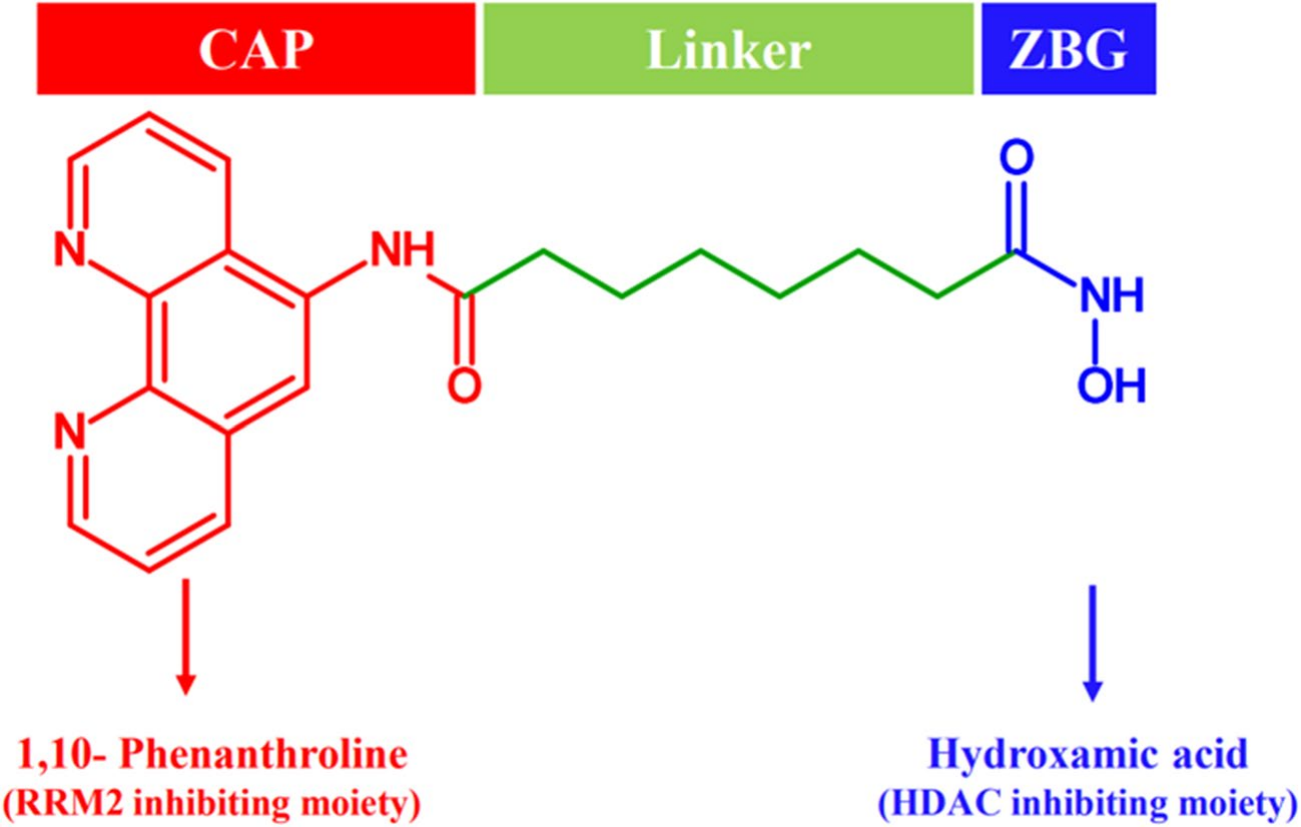
Design of PA as a novel dual HDAC/RR inhibitor

Considering the potential benefits of co-targeting HDACs and RR, the present work focuses on the design, evaluation, and discovery of a novel dual HDAC and RR inhibitor based on 1,10-phenanthroline and hydroxamic acid structural moieties. The chemical structure of the designed compound *N*^1^-hydroxy-*N*^8^-(1,10-phenanthrolin-5-yl)octanediamide (PA) is similar to SAHA (a known HDAC inhibitor), but features the substitution of the benzene moiety with the iron chelator 1,10-phenanthroline as shown in Fig. 1. PA's structural analogy to SAHA and the presence of an iron chelator, 1,10-phenanthroline, which could serve as a RR inhibiting moiety [25], positions it as a starting point for the systematic exploration and characterization as a novel dual HDAC/ RR inhibitor.

## Materials and methods

### General information

Solvents and reagents were procured from Sigma Aldrich (USA) and Finar (India). Reactions were monitored using TLC with silica gel 60 F254 plates, and melting points were determined with an EI capillary instrument. PA purity was assessed by Waters Alliance HPLC (USA) using a Waters C18 column with a 20:80 water-acetonitrile mobile phase. Mass spectrometry data was procured using Agilent 6520 Q-TOF (USA), while FTIR spectra were recorded on a Shimadzu FTIR-8310 (Japan). NMR spectra used a Bruker Ascend instrument (USA) with DMSO-d6 as the deuterated solvent.

### Chemistry

#### Synthesis of intermediate methyl-8-oxo-8-(1,10-phenanthrolin-5-ylamino) octanoate

In a 10 mL tetrahydrofuran solution, 1.50 mmol of 5-amino-1,10-phenanthroline and 1.94 mmol of methyl-8-chloro-8-oxooctanoate were mixed with 6.00 mmol of triethylamine, stirred vigorously at room temperature, and heated in a microwave at 150 °C for 10 min. After cooling, the ester-containing mixture was extracted with ethyl acetate [26].

Pale yellow solid; yield: 57.08%; mp: 86 °C; retention factor (R_f_): 0.93 (mobile phase: CHCl_3_/CH_3_OH; 9:3). HRMS (Q-TOF) m/z for C_21_H_23_N_3_O_3_, Calc. [M + H]^+^ 366.426 found. 366.185.

#### Synthesis of PA (N^1^-hydroxy-N^8^ -(1,10-phenanthrolin-5-yl) octanediamide)

A solution containing 53.50 mmol of hydroxylamine hydrochloride in methanol was refluxed until clear, and 53.50 mmol of potassium hydroxide in methanol was added to the above mixture and mixed at 0 °C. After cooling, a white solid formed was filtered, and the filtrate was added to 1.20 mmol of previously synthesized ester. The mixture was stirred for 24 h at room temperature. Upon completion, the pH was neutralized, and the product was extracted with ethyl acetate [26].

Beige solid; yield: 17.75%; mp: 200 °C; R_f_: 0.16 (mobile phase: CHCl_3_/CH_3_OH; 4:1). Purity > 95% (HPLC). FTIR (KBr, cm^−1^): 3199.91 (NH); 1695.43 (C = O); 1244.09 (C-N); 3500.80 (OH).^1^H NMR (400 MHz, DMSO-d6, ppm): *δ* 10.43 (s, 1H, hydroxamic OH); 10.27 (s, 1H, amide NH); 9.12 (d, 1H, *J* = 3.6 Hz); 9.03 (d, 1H,* J* = 4.0 Hz); 8.70 (s, 1H, amide NH); 8.66 (d, 1H, *J* = 8.4 Hz); 8.48 (d, 1H, *J* = 8.0 Hz); 8.18 (s, 1H); 7.85 (dd, 1H,* J* = 8.4 Hz, 4.4 Hz); 7.77 (dd, 1H, *J* = 7.6 Hz, 3.6 Hz); 2.53 (t, 2H, *J* = 8.0 Hz); 2.33 (t, 2H, *J* = 7.2 Hz); 1.69 (m, 2H); 1.54 (m, 2H); 1.37 (m, 4H), ^13^C NMR (100 MHz, DMSO-d6, ppm): *δ* 173.86, 173.09, 150.48, 149.85, 145.83, 143.69, 136.72, 132.97,132.52, 128.62, 125.22, 124.29, 123.51, 120.34, 36.30, 33.71, 28.86, 28.74, 25.57, 24.83. HRMS (Q-TOF) m/z for C_20_H_22_N_4_O_3_, Calc. [M + H]^+^ 367.413 found. 367.172.

### Biological evaluation

#### Cell culture

Human cancer cells (SiHa, HepG2, MCF7, Cal27) were obtained from ATCC, while foreskin fibroblast cells were generated at the Manipal School of Life Sciences. Cells were cultured in DMEM with 10% FBS, maintained in an Eppendorf CellXpert® C170 incubator at 37 °C with 5% CO_2_, and medium was renewed every 48–72 h.

#### Cell viability assay

Cancer cells (1 × 10^5^ cells/mL) and fibroblast cells (5 × 10^4^ cells/mL) were seeded in 96-well plates. Test compounds, dissolved in DMSO and diluted in DMEM with 10% FBS, were treated for 48 h. MTT (5 mg/mL) was added, and after 4 h, absorbance was measured at 570 and 630 nm using a VARIOSKAN™ 3001 multimode multiplate reader [27].

#### Cell cycle analysis

SiHA cells were synchronized through a 24 h period of serum deprivation, followed by a 48 h exposure to test compounds (IC_50_ and IC_70_) in DMEM with 10% FBS. After trypsinization, cells were fixed in 70% ethanol, treated with RNase-A, stained with propidium iodide, and analyzed using the Partec CyFlow Space with FloMax software (Germany) [28].

#### Apoptosis assay

SiHa cells were cultured at a density of 1 × 10^6^ cells/mL in 10 cm plates. Upon reaching 80% confluency, cells were subjected to 48 h treatment with test compounds (IC_50_ and IC_70_). Following two PBS washes, trypsinization, and suspension in Annexin V binding buffer, cells were incubated, stained with propidium iodide, and analyzed for apoptosis using Apoptosis Detection Kit I and Partec CyFlow Space with FloMax software (Germany) [29].

#### Intracellular reactive oxygen species (ROS) measurement

SiHa cells were cultured at a density of 1 × 10^6^ cells/mL in 10 cm plates. Upon reaching 80% confluency, the cells were subjected to 48 h treatment with test compounds at IC_50_ and IC_70_ concentrations. Post-treatment, cells were stained with 1 µg/mL DCFH-DA dye, incubated for 45 min at 37 ºC, and the fluorescence intensity was measured using Partec CyFlow Space and FloMax software [30].

#### Confocal microscopy

SiHa cells were grown on a coverslip treated with test compounds at IC_50_ and IC_70_ for 48 h, followed by 4% paraformaldehyde fixation for 10 min and treatment with 15 μg of phalloidin–tetramethylrhodamine B isothiocyanate for 30 min. After washing with PBS, cells were stained with Hoechst 33,342 (1 μg/mL), mounted on a glass slide, and imaged using a Leica SP8 confocal microscope with Leica application suite (Germany) [31].

#### Anchorage-dependent colony formation assay

SiHa cells (500 cells/well) were seeded in 6 cm plates. After 24 h, cells were treated with the IC_50_ concentration of test compounds and incubated for 21 days with media changes every three days. On day 21, colonies were stained with 0.4% crystal violet for 10 min, washed twice with PBS, and then counted [31].

#### In vitro cell migration assay

SiHa cells were cultured in 6-well plates using DMEM with 10% FBS until they formed a monolayer. A scratch was done on the cell monolayer by using a sterile 200 μL pipette tip. Subsequently, the cells were treated with the PA at IC_50_ concentration, and the extent of cell migration was assessed by analyzing the wound area at 0th, 24th, and 48th h time intervals using ImageJ software (Wayne Rasband, USA) [31].

#### In vitro HDAC assay

In a black 96-well plate, HDAC assay buffer, varied concentrations of test compounds, and SiHa cell nucleus extract were combined and incubated for 30 min. The enzymatic reaction commenced with the addition of the fluorescent substrate, Boc-Lys(Ac)-AMC, and lasted for 2 h at 37 °C. Reaction termination involved adding a developer, followed by an additional incubation at room temperature for 10–15 min. Fluorescence intensity was assessed using a VARIOSKAN™ 3001 multimode multiplate reader at an excitation wavelength of 354 nm and emission wavelength of 450 nm [32].

#### Western blot analysis

SiHa cells (2 × 10^5^ cells/well) were cultured in 6-well plates, treated with varied concentrations of test compounds for 48 h, and lysed in RIPA buffer. After centrifugation, protein content was quantified using Bradford assay. Protein extracts were subjected to SDS-PAGE and were transferred to the nitrocellulose membrane. Following blocking, membranes were probed with CST antibodies (Acetyl-Histone H3, B-Actin) at 1:3000 dilution and secondary anti-rabbit antibodies (1:5000 dilution). iBright™ CL1500 Imaging System captured the images.

#### Spectrophotometric determination of complex formation

Absorption measurements were carried out using a VARIOSKAN™ 3001 multimode multiplate reader. The experiments were performed at a wavelength from 200 to 700 nm.

#### Inhibition of DNA synthesis

SiHa cells (5 × 10^6^) were treated with varying test compound concentrations for 48 h. Following incubation, cells were pulsed with 5 nM ^3^H-thymidine for 30 min at 37 °C. After collection, washing, and total DNA extraction, radioactivity was quantified as counts per min (CPM) using a Tri-carb 2900TR liquid scintillation (Perkin Elmer, USA) [33].

#### dNTP pool analysis

SiHa cells were subjected to serum starvation for 24 h before treatment with PA at an IC_50_ concentration for 20 h. Harvested duplicate samples (1 million cells each) were washed with ice-cold PBS, and an extraction solution (6.5: 3.5 of acetonitrile and water mixture with 100 mM formic acid) was added. After incubation and centrifugation, the solution was lyophilized, reconstituted in 100 µL HPLC buffer A, and analyzed on a Waters Symmetry C18 column using HPLC. A stepwise gradient of Buffer A and B was employed, with detection at 260 and 280 nm, as mentioned by Crona et al., in 2016 [34]. Quantification was based on peak area measurements in the chromatogram, providing relative quantification of the analyzed components.

#### Partition coefficient studies

The partition coefficient (Kpart) of PA was determined in a 1-octanol/water system. 1-octanol was added to MOPS buffer containing PA (IC_50_), and OD at 276 nm was measured before and after each addition. Kpart values were calculated. [35].

### In silico analysis

#### Molecular docking

Molecular docking studies were conducted using Maestro version 11.4 (Schrodinger Inc.). The XP system in Maestro was employed for ligand–protein interaction analysis [36].

#### MD simulations

Molecular dynamic (MD) simulations were conducted using the Desmond module (Schrodinger Inc.). A three-step workflow included system setup, solvation with the simple point charge (SPC) solvent model, and a 100 ns simulation [36].

#### ADME prediction

The Swiss ADME (absorption, distribution, metabolism, and excretion) by the Swiss Institute of Bioinformatics was used for the ADME studies in PA.

#### Density function theory (DFT) and time-dependent density-functional theory (TD-DFT) analysis

The Fe^3+^ ion complexes with PA were optimized in two modes (a and b) using Becke's B3LYP functional, SVP basis set, and D3BJ damping scheme in hybrid DFT. The free molecule was optimized with a TZVP basis set and B3LYP functional. Single-point TD-DFT calculations were performed using ORCA3, and visualization utilized Avogadro software.

## Results and discussion

### Design of novel HDAC/ RR dual inhibitor

A 1,10-phenanthroline hydroxamate-based small molecule PA was previously reported and synthesized by Ye et al*.,* in 2013 [25] as the starting material for the synthesis of 1,10-phenanthroline hydroxamic acid-based ruthenium (II) polypyridyl based complexes. Substituting the benzene moiety of SAHA with 1,10-phenanthroline in PA suggested its potential as an RR inhibitor, given 1,10-phenanthroline's role as an iron chelator (a major class of RRM2/2B inhibitors) [37]. Additionally, prior reports on the synergistic cytotoxic effect of HU and 1,10-phenanthroline in combination with cancer cells further supported the investigation [38]. PA bearing a hydroxamic acid moiety, which is structurally analogous to HU, a known RR inhibitor, further prompted the evaluation of PA as a dual HDAC and RR (RRM2) inhibitor (Fig. 1) and its potential as an efficient anti-cancer agent through *in-vitro* assays and *in-silico* analysis.

### Chemistry

**Fig. 2:**
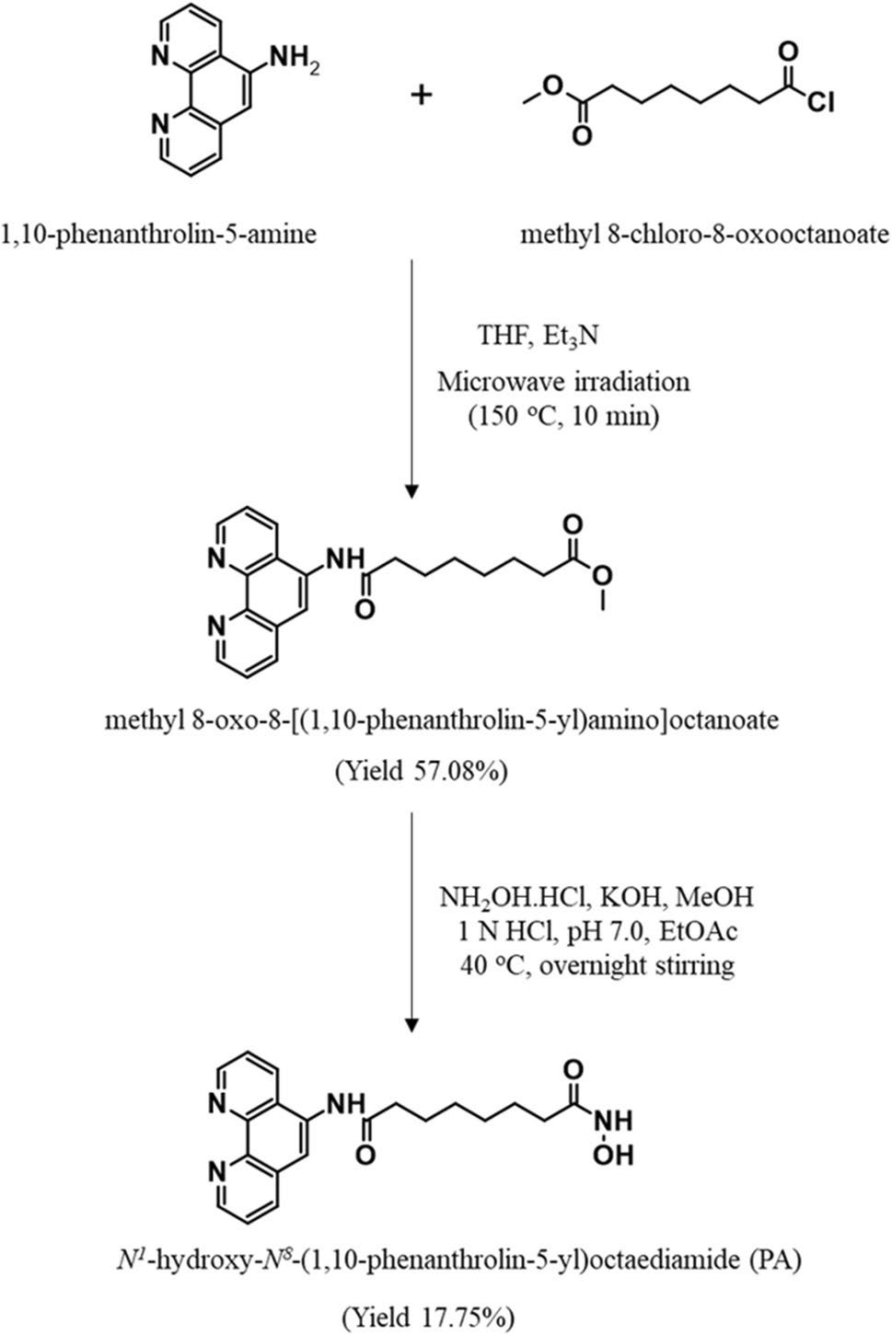
Reaction scheme for the chemical synthesis of **PA**

To synthesize the identified compound PA, Spencer et al*.,* in 2011, employed a modified version of the synthesis method [26]. The scheme (Fig. 2) involved a microwave-mediated reaction of 5-amino-1,10-phenanthroline with methyl-8-chloro-8-oxooctanoate, yielding an ester with a 57.08% yield. This ester was subjected to a coupling reaction with hydroxylamine to produce PA with a 17.75% yield. Characterization confirmed PA's structure using mass spectrometry, FTIR spectroscopy, and ^1^H and ^13^C NMR spectroscopy, while HPLC analysis and melting point calculation determined its purity.

### Biological evaluation

#### Cell viability assay

The MTT assay evaluated the cytotoxicity of the PA for 48 h in various human cancer cells (SiHa, HepG2, MCF-7, and Cal27) and normal human fibroblast cells. PA exhibited minimal cytotoxicity towards fibroblast cells, with an IC_50_ value exceeding 100 μM. Notably, PA demonstrated higher potency against SiHa cells (IC_50_: 16.43 μM) and Cal27 cells (IC_50_: 50.98 μM) while showing the least cytotoxicity towards HepG2 and MCF7 cells (Table 1).

**Table 1:** IC_50_ values of tested compounds

Compound	Antiproliferative activity IC_50_ (µM)^a^				
	SiHa	HepG2	MCF7	Cal27	Fibroblast
PA	16.43 ± 4.71	> 100	> 100	50.98 ± 3.08	> 100
1,10-Phenanthroline	42.86 ± 2.53	> 100	48.76 ± 11.97	53.00 ± 2.18	> 100
HU	> 100	> 100	> 100	> 100	> 100
SAHA	25.16 ± 3.69	61.9 ± 1.47	> 100	09.42 ± 4.14	> 100
DFO	71.52 ± 5.02	> 100	> 100	46.02 ± 3.07	> 100

^a^Data are represented as IC_50_ values (mean ± SD)

#### Cell cycle analysis

**Fig. 3:**
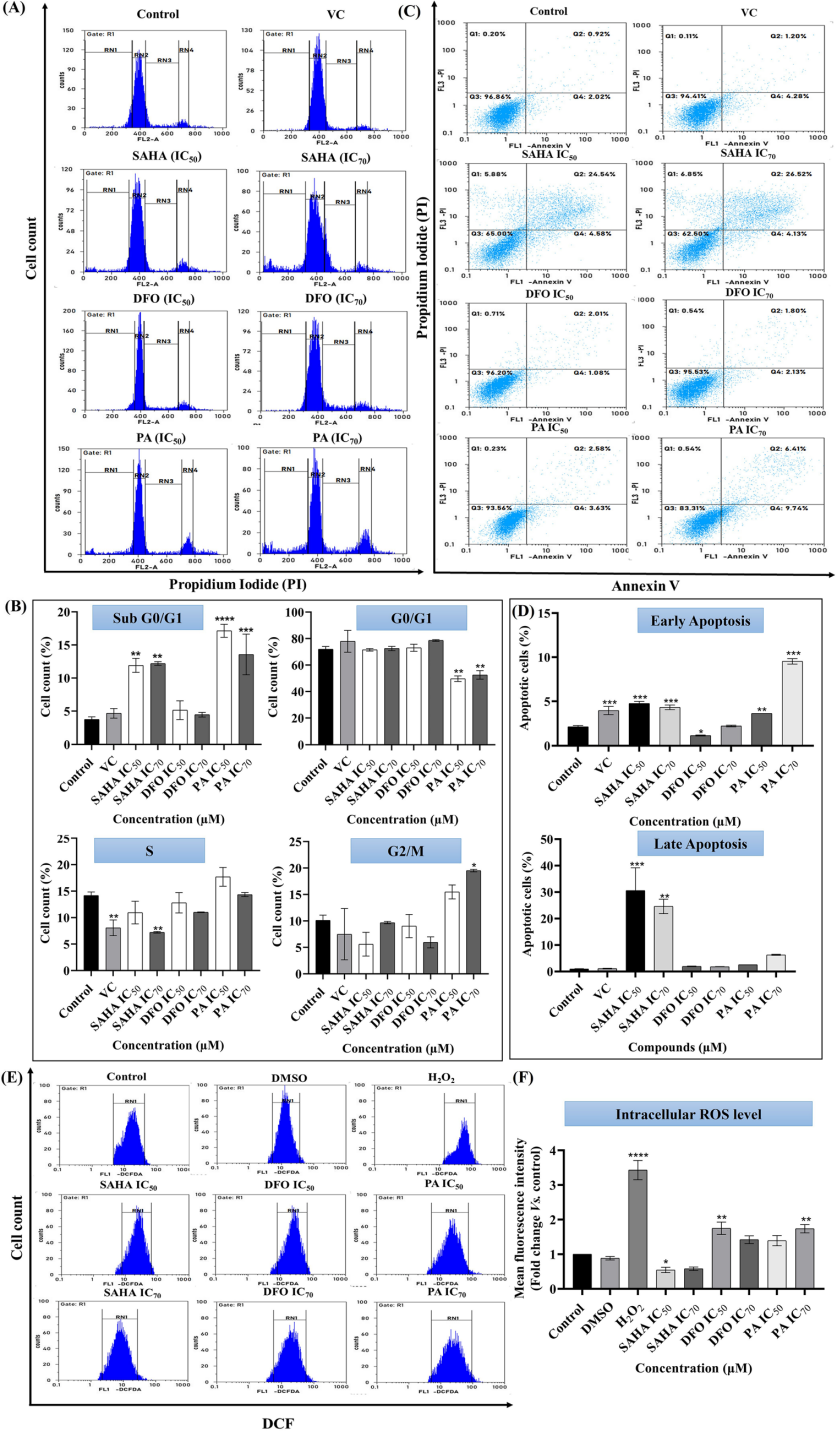
Cell cycle analysis, apoptosis and ROS at IC_50_ and IC_70_ concentrations of SAHA, PA and DFO for 48 h in SiHa cells **(A)** Cell cycle distribution (RN1: sub-G1/G0, RN2: G0/1/; RN3:S and RN4: G2/M), **(B)** Quantitative analysis of the treated groups compared to the control and vehicle control (VC) DMSO (0.1%) at different phases of the cell cycle. *p < 0.05, **p < 0.01, ***p < 0.001, ****p < 0.0001 (*Vs*. the control) determined with Student's t-test), **(C)** Apoptotic cell distribution, **(D)** Quantitative analysis of the treated groups compared to the control and VC at early and late apoptotic phase. *p < 0.05, **p < 0.01, ***p < 0.001 (*Vs*. the control) determined with Student's t-test, **(E)** Intracellular ROS distribution and **(F)** Quantitative analysis of the treated groups compared to the control and VC in the induction of intracellular ROS. *p < 0.05, **p < 0.01, ****p < 0.0001 (*Vs*. the control) determined with Student's t-test

The cytotoxicity of PA in SiHa cells was further explored by investigating its impact on cell cycle progression. PA treatment at IC_50_ concentration for 48 h increased the cell population at the S phase and a concentration-dependent elevation in the G2/M phase. Notably, PA also significantly arrested cells in the sub-G0/G1 phase, surpassing the effects induced by SAHA (Fig. 3A and B). These findings underscore the potential of PA to modulate cell cycle dynamics and induce cell death.

#### Apoptosis assay

The induction of apoptosis by PA at IC_50_ and IC_70_ concentrations over 48 h was further investigated. A notable surge in apoptotic cell percentages emerged in both PA and SAHA treatment groups. PA exhibited a distinct ability to induce early apoptosis (3.63% and 2.58% at IC_50_ and IC_70_ concentrations, respectively) and late apoptosis (9.53% and 6.28% at IC_50_ and IC_70_ concentrations, respectively) (Fig. 3C and D). While SAHA demonstrated overall higher apoptosis induction, PA stood out for its significant induction of early apoptosis compared to SAHA.

#### Intracellular reactive oxygen species (ROS) measurement

Upon treatment with compounds at their IC_50_ and IC_70_ concentrations for 48 h, the measurement of intracellular ROS levels using the fluorescent probe DCFH-DA revealed a significant increase induced by both DFO and PA in SiHa cells. Compared to the control, PA exhibited fold changes of 1.39 and 1.75 at IC_50_ concentration and 1.74 and 1.42 at IC_70_ concentrations, respectively (Fig. 3E and F). These results suggest that PA-mediated growth inhibition and apoptosis in SiHa cells are associated with ROS generation.

#### Cell morphology

**Fig. 4:**
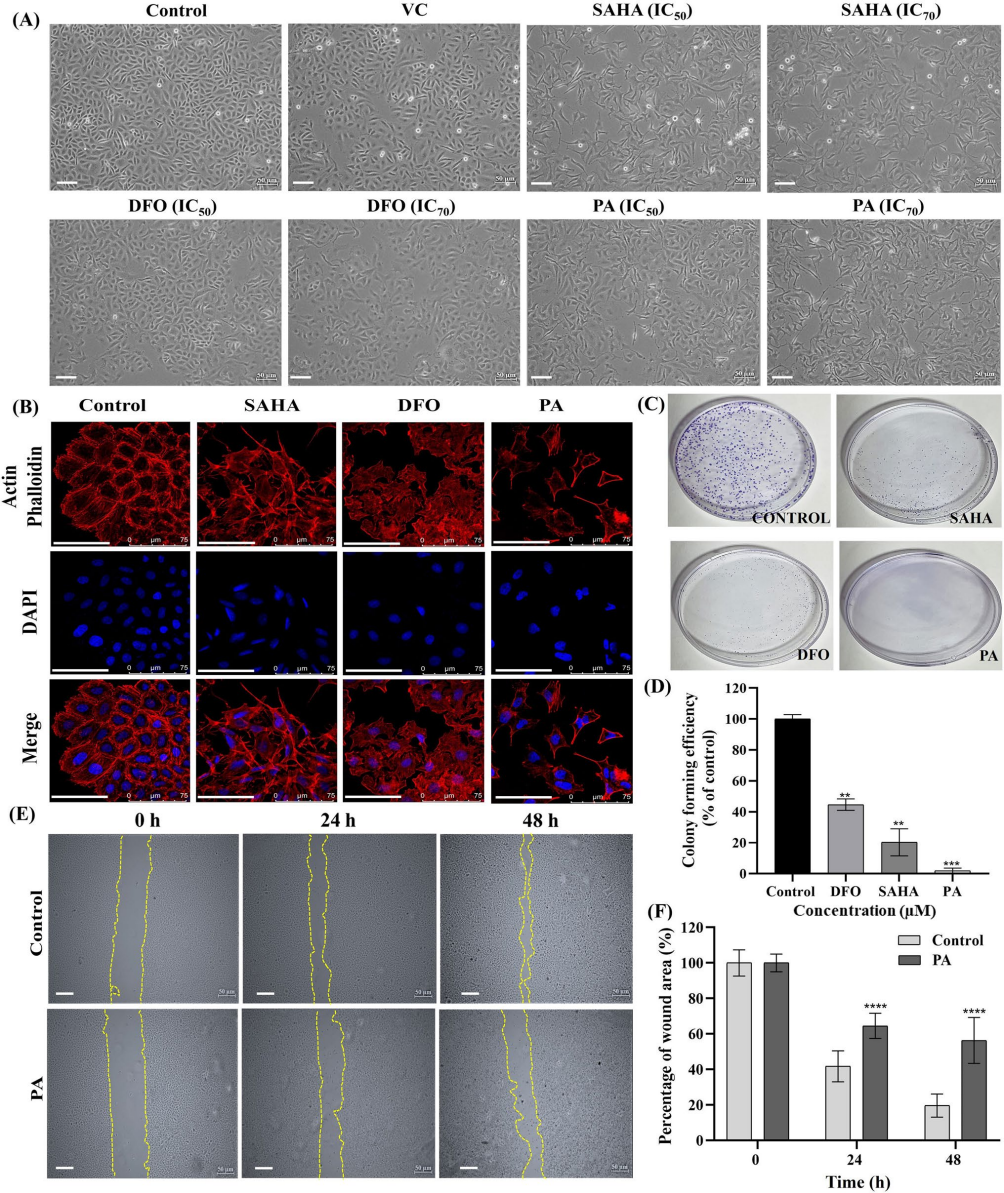
**(A)** The cellular morphology of SiHa cells upon treatment with VC (DMSO 0.1%), SAHA, DFO, and PA at IC_50_ and IC_70_ concentration for 48 h (scale bars: 50 μm), **(B)** The nuclear and cytoskeleton-stained images [actin fibers (red-stained), nuclei (blue-stained)] of the SiHa cells at IC_50_ concentration of SAHA, DFO, and PA for 48 h (scale bars: 75 μm), **(C)** The SiHa cell colonies at IC_50_ concentration of SAHA, DFO, and PA for 48 h, **(D).** Quantitative analysis of colony formation of compounds at their respective IC_50_ values. [**p < 0.01, ***p < 0.001 (*Vs*. the control)] determined with Student's t-test, **(E)** The migration of SiHa cells at various time intervals (0, 24, and 48 h) at IC_50_ concentration of PA (scale bars: 50 μm) and **(F)** Quantitative analysis of the cell migratory effect upon PA treatment. ****p < 0.0001. (*Vs.* the control) determined with Student's t-test

Optical bright-field images of SiHa cells treated with test compounds at their IC_50_ and IC_70_ concentrations for 48 h were captured. These images revealed that the treatment with PA-induced a distinct morphological alteration in cells, similar to the effects observed in SAHA-treated cells (Fig. 4A).

#### Confocal analysis

Further, the phenotypic characteristics of cells treated with test compounds at their respective IC_50_ concentrations for 48 h were better studied using actin-phalloidin, a specific F-actin fluorescent probe, and DAPI, a nuclear stain. These stains allowed us to examine whether the cytoskeleton and nuclei were targets of PA treatment in SiHa cells. The results suggest that, compared to the control, SiHa cells treated with test compounds (SAHA, DFO, and PA) failed to maintain overall actin filament structures. Moreover, PA-treated cells exhibited a similar change in their actin filament structures to that observed with SAHA, as shown in Fig. 4B. These findings indicate that PA-induced apoptosis could be associated with the disruption of F-actin microfilaments.

#### Colony formation assay and inhibition of cell migration

Upon treating SiHa cells with compounds at their IC_50_ concentrations for 48 h, significant reductions in clonogenic efficiency were observed, with PA exhibiting the most potent inhibition (1.99%), compared to SAHA (20.32%) and DFO (44.63%) (Fig. 4C and D). In the migration assay, the PA-treated group showed a higher wound area percentage (56.33%) at 48 h post-wounding, surpassing the control group (19.64%) (Fig. 4E and F). These findings emphasize PA's efficacy in hindering colony formation and migratory properties of SiHa cells at its IC_50_ concentration, surpassing the effects of SAHA and DFO at their respective IC_50_ concentrations.

#### In vitro HDAC inhibition studies

**Fig. 5:**
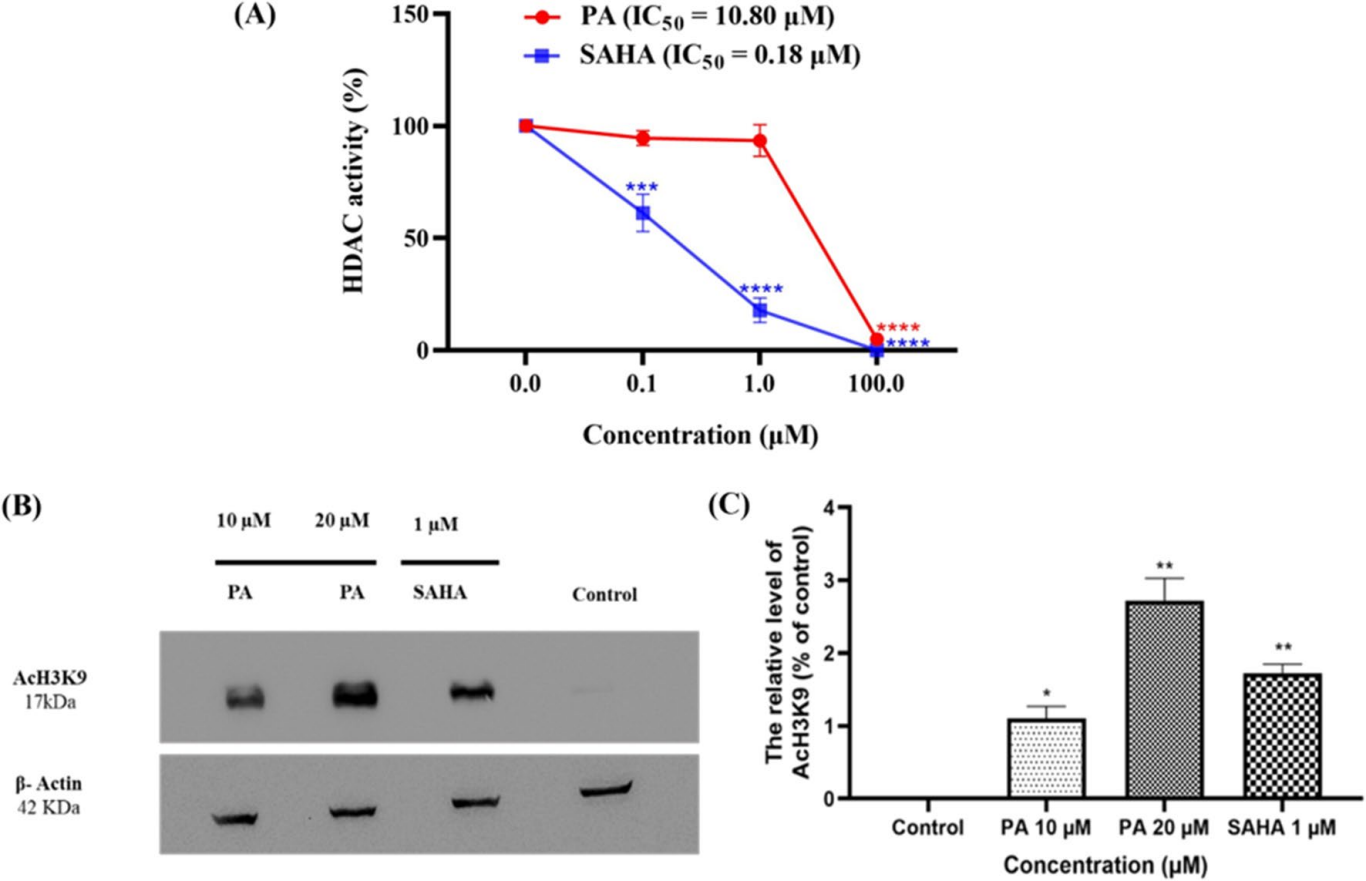
**(A)** HDAC activity of PA and SAHA in SiHa cells, **(B)** Western blot analysis to determine the effect of compound PA on the acetylation at H3K9 in SiHa cells treated with PA for 48 h, and **(C)** Quantification of PA-mediated H3K9 hypoacetylation by densitometry. *p < 0.05, **p < 0.01 (*Vs.* the control) determined with Student's t-test

The HDAC inhibitory activity of SAHA and PA was assessed using a fluorescent-based assay, employing SiHa cell nuclear extracts as the HDAC source and a fluorogenic acetylated histone peptide fragment as the substrate. Both exhibited inhibitory effects on HDACs. SAHA, with an IC_50_ value of 0.18 µM, demonstrated a more potent inhibitory effect on HDACs compared to PA (IC_50_, 10.80 µM) (Fig. 5A).

#### Effect of PA on histone hyperacetylation

The western blotting assays, with β–actin as a negative control, demonstrated that exposure of SiHa cells to varying concentrations of PA (10 and 20 µM) for 48 h resulted in a dose-dependent increase in acetylated Histone H3 levels. This effect closely mirrored the impact observed with the established HDAC inhibitor SAHA (1 µM) (Fig. 5B and C). The findings confirm that PA effectively inhibits the deacetylase activities of HDACs, leading to histone H3 acetylation.

#### Iron chelation studies

**Fig. 6:**
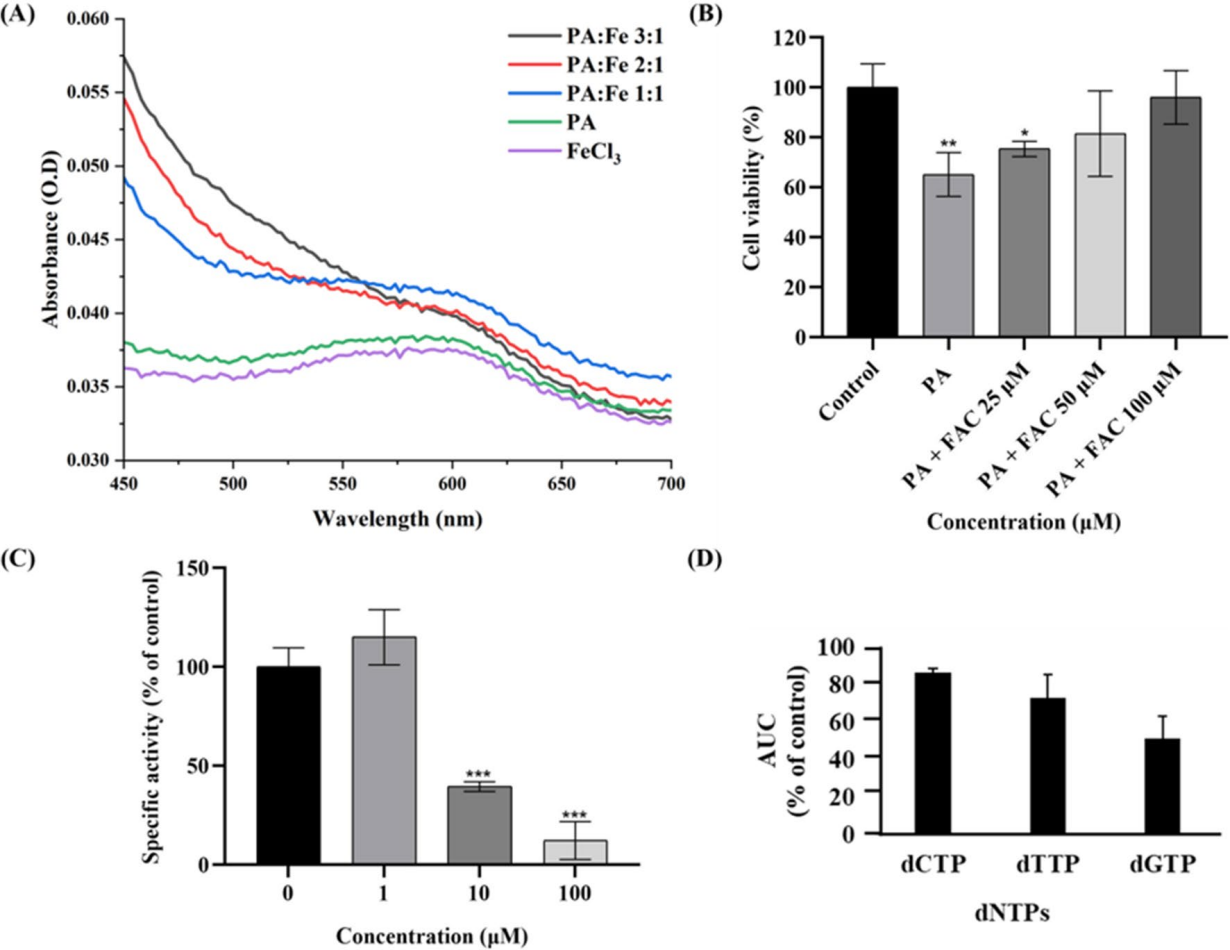
**(A)** Absorbance spectrum of PA-Fe^3+^ complex at three different concentration ratios (3:1, 2:1, and 1:1) respectively, with absorbance maximum centered between 500–600 nm, **(B)** Cell viability of SiHa when treated in combination with variable FAC concentrations and a constant PA concentration (IC_50_). *p < 0.05, **p < 0.01 (*Vs*. the control) determined with Student's t-test; Impact of PA on RR activity and cellular dNTP pools**, (C)** Influence of PA on 3H-thymidine incorporation and **(D)** Quantification of dNTPs in PA-treated cells at their IC_50_ values. ***p < 0.001 (*Vs*. the control) determined with Student's t-test

To check PA's binding capacity to Fe^3+^ ion, the complex formation between the Fe^3+^ ion and PA was investigated using absorbance spectroscopy. Figure 6A shows the absorption spectrum of the PA-Fe^3+^ complex at different concentration ratios (PA: Fe^3+^; 3:1, 2:1, and 1:1) using a VARIOSKAN™ 3001 multimode multiplate reader from ThermoFisher Scientific, Finland.

#### Measurement of the partition coefficient (water/octanol)

The hydrophobicity of PA, assessed by log (Kpart7.4), was determined as 0.82, closely aligning with the in silico Schrodinger analysis tool result of 1.098. These findings suggest that PA has the potential to preferentially accumulate in an *N*-octanol fraction over an aqueous fraction, indicating characteristics conducive to cell membrane penetration distinguishing it from typical metal ion chelators.

#### Suppression of cytotoxicity by iron supplementation in SiHa

To confirm the intracellular Fe^3+^ chelation ability of PA, three different concentrations (25, 50, 100 µM) of ferric ammonium citrate (FAC) with PA (IC_50_) were co-administered in SiHa cells, and the cell viability was assessed using MTT after 48 h. Starting at a concentration of 25 µM, FAC reduced PA cell toxicity in a concentration-dependent manner, eliminating the inhibitory function of PA (Fig. 6B).

#### Effects of PA on RR Activity and dNTP pool

To measure the impact of PA on the enzymatic activity of RR, the incorporation of ^3^H-labeled thymidine into the DNA of SiHa cells was determined. ^3^H-thymidine incorporation into DNA was significantly decreased in PA treated group (48 h treatment) in comparison to control values in a concentration-dependent manner with an IC_50_ value of 9.34 µM (Fig. 6C). PA treatment also caused a marked decrease in intracellular dNTP levels (Fig. 6D).

### In silico analysis

#### Molecular docking

**Fig. 7:**
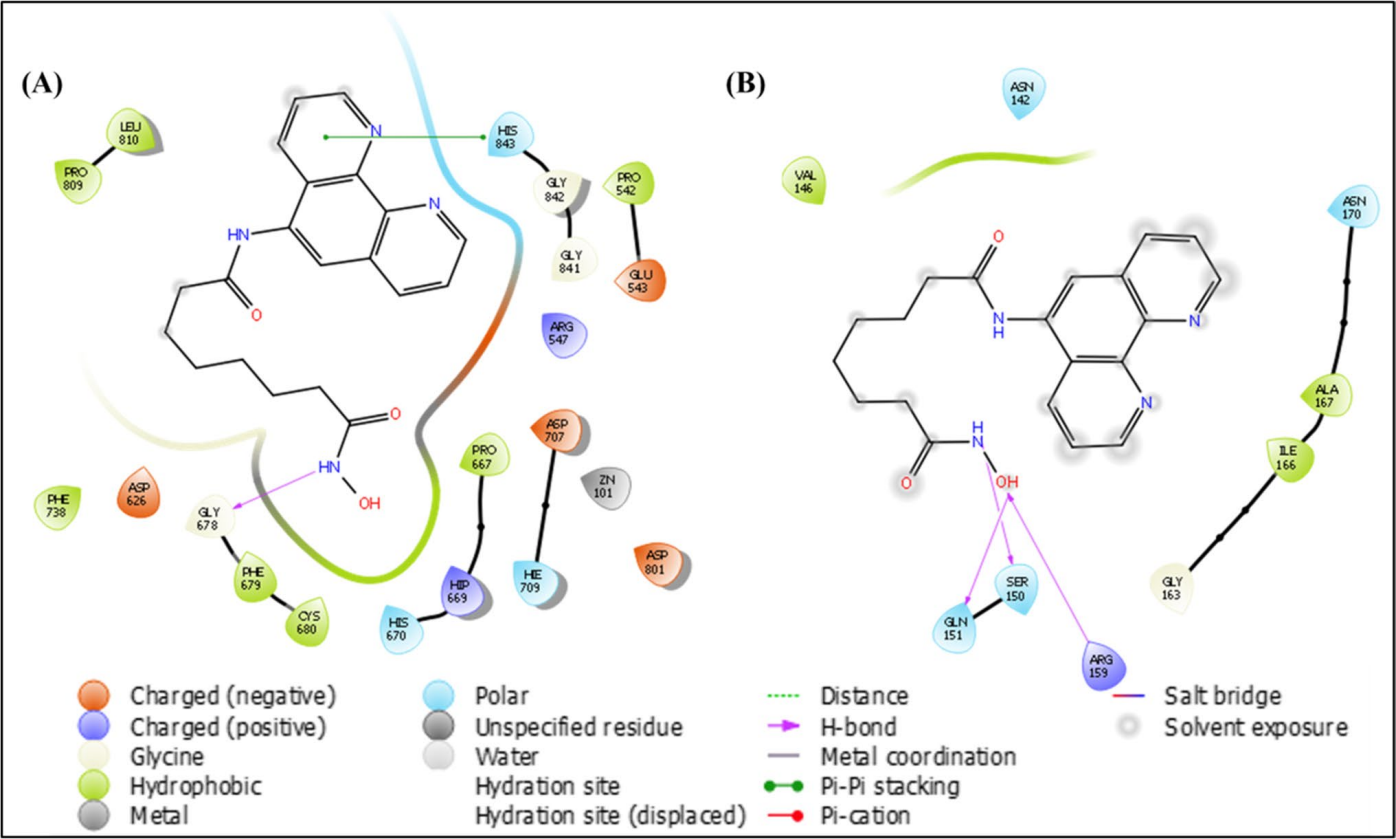
Molecular docking studies **(A).** Predicted 2D binding mode and receptor–ligand interaction diagrams of PA with HDAC7. The crystal structure of the protein was obtained from the Protein Data Bank (PDB ID: 3ZNR) and **(B).** Predicted 2D binding mode receptor–ligand interaction diagrams of PA with RRM2. The crystal structure of the protein was obtained from the Protein Data Bank (PDB ID: 2UW2)

In silico molecular docking studies were conducted using Maestro version 11.4 (Schrodinger Inc.) to investigate the interaction of PA with classical HDACs (class I, II, and IV) and RRM2. PA exhibited interactions with all isoforms of classical HDACs, displaying the highest binding affinity with HDAC7 (-9.633 kcal/mol). While its binding affinities toward HDAC7 and HDAC8 were higher than SAHA, its docking with RRM2 revealed lower affinity (-3.681 kcal/mol) compared to DFO (-6.549 kcal/mol) as shown in Table 2. The 2D interaction data of these complexes, as shown in Fig. 7A and B, include metal ion, polar and hydrophobic interactions (Supplementary file number 1, Table S1). These findings support PA as a potential dual HDAC and RR inhibitor.

**Table 2:** XP dock scores of the compounds with all the classical HDAC isoforms and RRM2

Compound	HDAC1 (4BKX)	HDAC2 (4LY1)	HDAC3 (4A69)	HDAC4 (2VJQ)	HDAC5 (Q9UQL6)	HDAC6 (3PHD)	HDAC7 (3ZNR)	HDAC8 (1T69)	HDAC9 (Q9UKV0)	HDAC10 (6UII)	HDAC11 (Q96DB2)	RRM2 (2UW2)
PA	-4.739	-8.225	-2.972	-7.935	-5.103	-4.666	-9.633	-9.386	-4.652	-8.735	-7.894	-3.681
1,10- Phenanthroline	-4.065	-4.150	-2.208	-3.139	-3.037	-4.612	-4.135	-3.397	-2.198	-4.311	-5.241	-
HU	-2.541	-6.824	-3.301	-5.728	-3.042	-3.051	-5.466	-7.602	-4.153	-6.355	-5.885	-3.782
SAHA	-4.430	-12.036	-1.183	-8.286	-1.166	-3.517	-8.244	-9.908	-2.237	-8.966	-7.734	-
DFO	-	-	-	-	-	-	-	-	-	-	-	-6.549

#### Molecular dynamic stimulation

In this investigation, molecular dynamics simulations were conducted for the docked structures of compounds SAHA, PA, and the co-crystallized compound TMP269 with HDAC7 (PDB—3ZNR), as well as PA, HU, and DFO with RRM2 (PDB—2UW2), with each simulation generating 1000 trajectories. The stability of the protein–ligand complexes was assessed using Root Mean Square Deviation (RMSD) values, providing insights into their structural integrity over time, as shown in supplementary file number 1, Fig. S1 and S2. Further, the detailed interactions in the protein–ligand complexes are summarized in supplementary file number 1, Table S2.

#### ADME prediction

PA exhibited favorable human GI absorption, indicating the potential to traverse biological membranes with high bioavailability in the GIT. All the test compounds adhered to Lipinski's rule of five, except DFO, violating the molecular weight parameter (> 500 g/mol), as shown in Supplementary file number 1, Table S3.

#### DFT and TD-DFT analysis

**Fig. 8:**
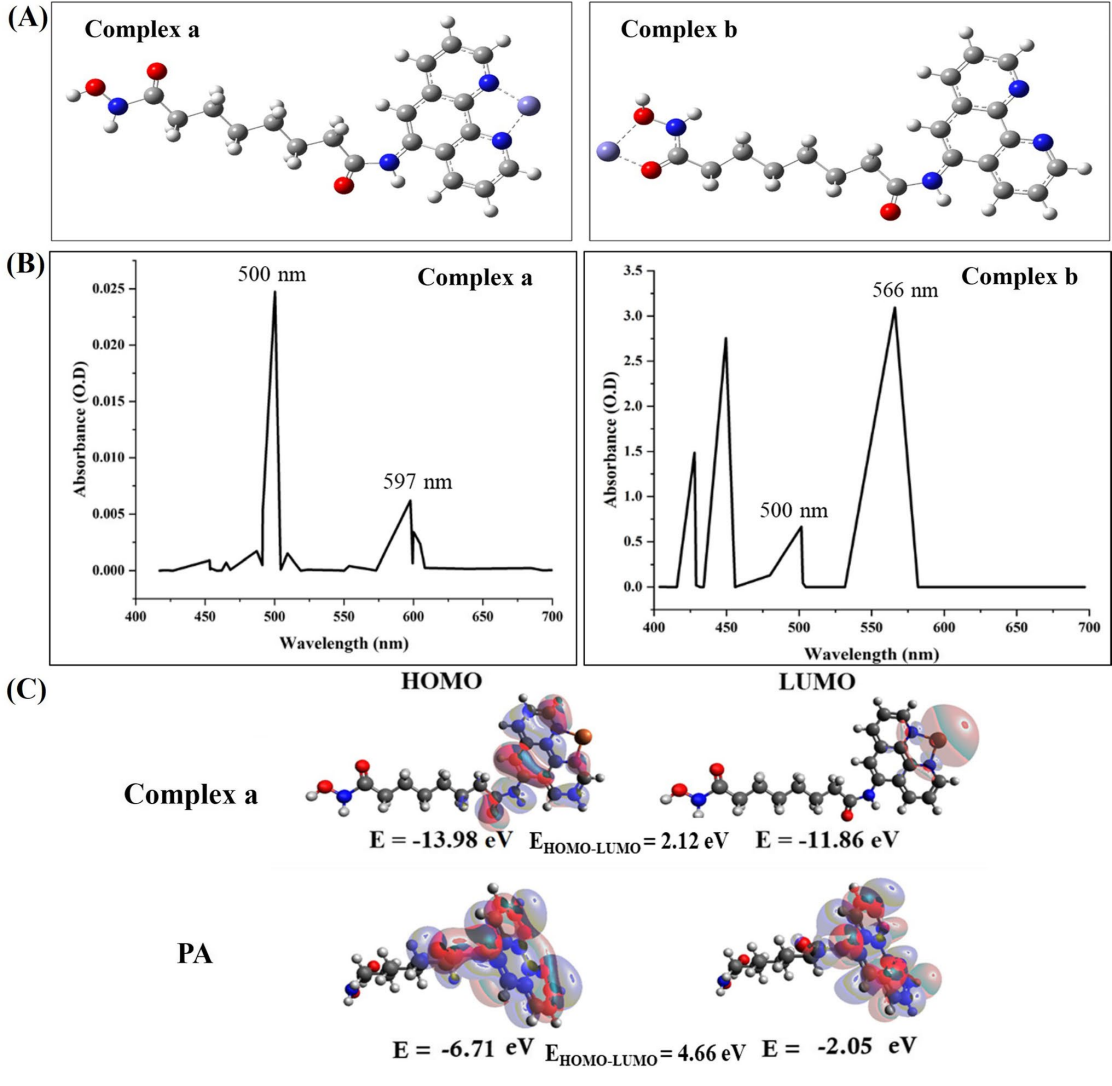
**(A).** Two different modes of complexation of Fe^3+^ ion with PA (complex **a** and **b**)**; (B).** Predicted absorption spectra of the two complexes of Fe^3+^ iron with PA with absorbance maximum centered at 500 and 597 nm (complex **a**) *&* 500 nm and 566 nm (complex **b**) and **(C).** Molecular orbitals of the complex a of Fe^3+^ with PA and free molecule PA using TD-DFT method at B3LYP/SVP level of theory

The DFT analysis revealed that complex **a**, a potential iron complex of PA, exhibits greater stability than complex **b** (Fig. 8A), indicating a preference for the Fe^3+^ ion to form a complex with the nitrogen atoms in 1,10-Phenanthroline. This aligns with the hypothesis that PA forms a Fe^3+^ ion complex through 1,10-Phenanthroline, potentially contributing to the inhibition of RR activity. To confirm complex formation, TD-DFT calculations for complexes **a** and **b**, along with free PA, were conducted. TD-DFT predicted absorption bands at 500 and 597 nm (complex **a**) and 500 and 566 nm (complex **b**), resembling experimental spectra (Fig. 8B). While both PA-iron complexes were stable, 1,10-Phenanthroline's iron-binding affinity favored complex **an** over hydroxaminic groups in HU, lacking such affinity. Therefore, complex** a** emerges as the probable formation based on these considerations. The TD-DFT canonical molecular orbitals and related energies for complex **a** and PA are depicted in Fig. 8C. HOMO and LUMO analysis provide insights into reactivity and kinetic stability. Complex exhibits less stability than PA, evidenced by a reduced HOMO–LUMO gap, suggesting increased chemical reactivity. Compounds with higher reactivity often exert larger inhibitory effects. Complex **a**, with HOMO and LUMO values of -13.98 eV and -11.86 eV, respectively, has a band gap of 2.21 eV. In contrast, PA's energy gap is 4.66 eV, with HOMO and LUMO energies of -6.71 eV and -2.05 eV, respectively. These results collectively imply that the RR enzyme is more effectively inhibited by the complex formed between PA and the Fe^3+^ ion in the enzyme (Fig. 8C).

## Discussion

The challenge of cancer resistance to chemotherapy has prompted a shift towards more potent anti-cancer drugs using a multi-target approach using hybrid drug molecules. These hybrids, engaging multiple targets, are considered more effective in addressing the complexity of cancer cells. While HDAC inhibitors are potential candidates for hybrid drug development due to their chemically flexible structures, there are currently no FDA-approved multi/dual-targeting drugs based on HDAC inhibitors. Nevertheless, ongoing research in preclinical and various clinical trial phases focuses on the development of HDAC-based hybrids. Notably, the synthesis of novel Schiff bases (uracil-linked) as dual inhibitors, specifically targeting type II HDAC, stands out as a crucial strategy in advancing cancer therapeutics. Similarly, the development of a first-in-class anti-cancer dual HDAC2/FAK inhibitor featuring hydroxamates/benzamides capped by pyridinyl-1, 2, 4-triazoles further emphasizes the importance of simultaneous targeting of multiple pathways critical for tumor growth. The incorporation of unique structural motifs enhances selectivity and bioavailability, and the apoptotic potential of these compounds highlights their therapeutic relevance. Overall, the pursuit of dual HDAC inhibitors reflects a paradigm shift in cancer treatment, emphasizing the necessity of comprehensive strategies to effectively combat the complexity of cancer biology [39, 40]. Therefore, much effort is needed to develop novel and potent HDAC-based hybrid drugs [41–43].

The current study introduces a 1,10-phenanthroline hydroxamic acid derivative, referred to as PA within this research as a novel dual HDAC/RR inhibitor with enhanced anticancer activity. The chemical structure of PA, resembling that of SAHA but with the substitution of the benzene moiety by the known intracellular iron chelator 1,10-phenanthroline, positions it as an interesting anticancer candidate for investigation. Numerous studies have highlighted synergistic anti-cancer activities resulting from the combination of HDACs and RR inhibitors. The presence of both 1,10-phenanthroline and hydroxamic acid moieties within the structure of PA suggests its potential for dual inhibitory effects on both RRM2 and HDACs, respectively. Also, the hydroxamic acid moiety in PA exhibits structural similarity to HU, a recognized class of RR (RRM2) inhibitor. Moreover, a previous research study has documented the synergistic cytotoxicity of HU and 1,10-phenanthroline in combination against cancer cells, providing further support for the potential and enhanced anti-cancer properties of PA. These collective characteristics provide a strong rationale for investigating PA as a potential anticancer dual inhibitor of HDAC and RR. It is worth noting that substituted derivatives of a molecule may exert diverse impacts on target enzymes. However, in this study, the selection of PA over other substituted derivatives is based on its status as a previously synthesized and reported compound with well-established properties. Therefore, choosing PA for investigation as a novel dual HDAC/RR inhibitor serves as a strategic starting point for the systematic exploration of its anticancer biological activities. This selection establishes a solid foundation for future findings, wherein structural derivatives based on PA may lead to the discovery of novel HDAC/RR inhibitors with potentially even more enhanced anticancer activities.

Solid tumors often display epigenetic abnormalities, such as aberrant histone deacetylation, resulting in the suppression of tumor suppressor genes and activation of oncogenes. As previously mentioned, by promoting histone acetylation and chromatin relaxation, HDAC inhibitors can counteract these changes. This mechanism can heighten the responsiveness of solid tumors to other therapeutic agents, potentially leading to improved treatment outcomes [44]. While HDAC inhibitors show significant promise, resistance to them is frequently encountered. Moreover, HDAC inhibitors have demonstrated limited efficacy in the treatment of solid tumors. Evidently, they have primarily gained FDA approval for hematological cancers, with restricted effectiveness against solid tumors when administered as standalone treatments. However, it is reported that combination therapy involving HDAC inhibitors has exhibited promising anticancer effects in both preclinical and clinical investigations in both solid and hematological malignancies [45]. Building on this background, our study focuses on the development of PA, a compound that concurrently inhibits HDAC and RR. We aim to address this research gap by assessing the efficacy of our compound on solid tumor cell lines expressing HDAC7 and RRM2. These include SiHa (human cervical cancer cells), HepG2 (human liver cancer cells), MCF7 (human breast cancer cells), and Cal27 (human head and neck carcinoma), for biological assessments. The selection of some of these solid tumor cell lines is based on their expression of HDAC7 and RRM2, as reported by the Protein Atlas database [46/].

The in vitro biological evaluation of PA demonstrated significant cytotoxicity towards SiHa cells with minimal impact on normal fibroblast cells. The cell cycle analysis revealed sub-G1/G0 and G2/M phase arrest, indicating PA's potential to induce apoptosis. Annexin V/PI staining confirmed apoptosis as the primary mechanism of cell arrest. The concentration-dependent ROS accumulation in PA-treated groups aligns with previous research suggesting the selective action of HDAC inhibitors against cancer cells through the thioredoxin–redox system [47]. The unique structural motifs of PA not only influenced cellular morphology but also effectively suppressed malignant behaviors of cancer cells, including reduced migration and colony-forming efficiency. PA exhibited a dual inhibition by significantly reducing HDAC activity and forming a complex with Fe^3+^ ion, leading to a reduction in RR activity. In overall, the effective induction of cell death by PA in SiHa cells could be attributed to their elevated levels of HDAC7 and RRM2 compared to HepG2, MCF7, and Cal27 cells. Further, *in s ilico* docking studies supported the binding ability of PA, particularly towards HDAC7 and moderately towards RRM2 protein.

## Conclusion

In conclusion, the in vitro and in silico results in the present study support PA as a potential dual inhibitor targeting HDACs and RR, showcasing its cytotoxic impact on cervical cancer cells through ROS-mediated apoptosis. This study characterizes PA as a novel dual HDAC/RR inhibiting molecule, serving as a foundation for subsequent lead optimization studies to improve its anti-cancer efficacy.

### Supplementary Information

Below is the link to the electronic supplementary material.

Supplementary file1 (DOCX 700 KB)

Supplementary file2 (DOCX 759 KB)

## Data Availability

The data supporting the findings of this study are available within the supplementary materials available at 10.1007/s40199-024-00514-1.
