# Supplementary material for: Evaluation of 1,10-phenanthroline-based hydroxamate derivative as dual histone deacetylases/ribonucleotide reductase inhibitor with antitumor activities
Source: Daru. 2024 Apr 29;32(1):263–78. doi: 10.1007/s40199-024-00514-1 (PMC11087398; doi:10.1007/s40199-024-00514-1)
Supplement: Supplementary file 1 — Supplementary file1 (DOCX 700 KB) [file 40199_2024_514_MOESM1_ESM.docx]

**Supplementary file 1**

***Article title:***

Evaluation of 1,10-Phenanthroline-Based Hydroxamate Derivative as Dual Histone Deacetylases/ Ribonucleotide Reductase Inhibitor with Antitumor Activities

***Journal name:***

DARU Journal of Pharmaceutical Sciences

***Author names:***

Manasa Gangadhar Shetty^1^, Padmini Pai^1#^, Bipasa Dey^1#^ Kapaettu Satyamoorthy^2^, Suranjan Shil^3^, Usha Yogendra Nayak^4^, Aswini T^4^, Babitha Kampa Sundara^1*^.

***Affiliation:***

^1^Department of Biophysics, Manipal School of Life Sciences, Manipal Academy of Higher Education, Manipal - 576104, Karnataka, India

^2^Shri Dharmasthala Manjunatheshwara (SDM) University, Manjushree Nagar, Sattur, Dharwad - 580009, Karnataka, India

^3^Department of Chemistry, Manipal Centre for Natural Sciences (Centre of Excellence), Manipal Academy of Higher Education, Manipal - 576104, Karnataka, India

^4^Department of Pharmaceutics, Manipal College of Pharmaceutical Sciences, Manipal Academy of Higher Education, Manipal, Karnataka 576104, India

***e-mail address of the corresponding author.***

*babitha.ks@manipal.edu

*3.4.1 Molecular Docking*

**Table S1:** Summary of the ligand-protein interactions in XP docking analysis.

| **Ligand-protein complex** | **H-bond** | **Pi-Pi interaction** | **Hydrophobic interaction** | **Polar interaction** | **Metal interaction** | **Other interaction** |
| --- | --- | --- | --- | --- | --- | --- |
| PA-HDAC7 | Gly678 | His843 | Pro542, Pro667, Phe679, Cys680, Phe738, Leu810, Pro809, His843 | His670, Hie709, His843 | Zn101 | Positive: Glu543, Asp626, Asp707, Asp801; Negative: Arg547, Hip669 |
| PA-RRM2 | Ser150, Gln151, Arg159 | - | Val146, Ile166, Ala167 | Asn142, Ser150, Gln151, Asn170 | - | Negative: Agr159; Gly163 |

*3.4.2 MD Simulation*

The TMP269-HDAC7 complex displayed overall stability with slight drift from 30 to 100 ns (**Fig. S1A**). RMSD values for SAHA-HDAC7 demonstrated consistent stability throughout the simulation (Fig. S1C), while PA-HDAC7 exhibited continuous stability from 60 ns onward (Figure S1E). Despite initial drift in the interaction until 60 ns, PA-HDAC7 displayed improved interaction from 60 to 100 ns (**Fig. S1E**). The HU-RRM2 complex exhibited stable interactions with minimal drift from 35 to 49 ns (**Fig. S2A**), while DFO-RRM2 displayed interactions at 47 ns, 55 to 60 ns, and 74 to 100 ns (**Fig. S2C**). While both HU and DFO exhibited variations in stability, PA demonstrated superior stability throughout the simulation with slight drift from 4 to 10 ns, 60 to 75 ns, and 88 to 100 ns (**Fig. S2E**).


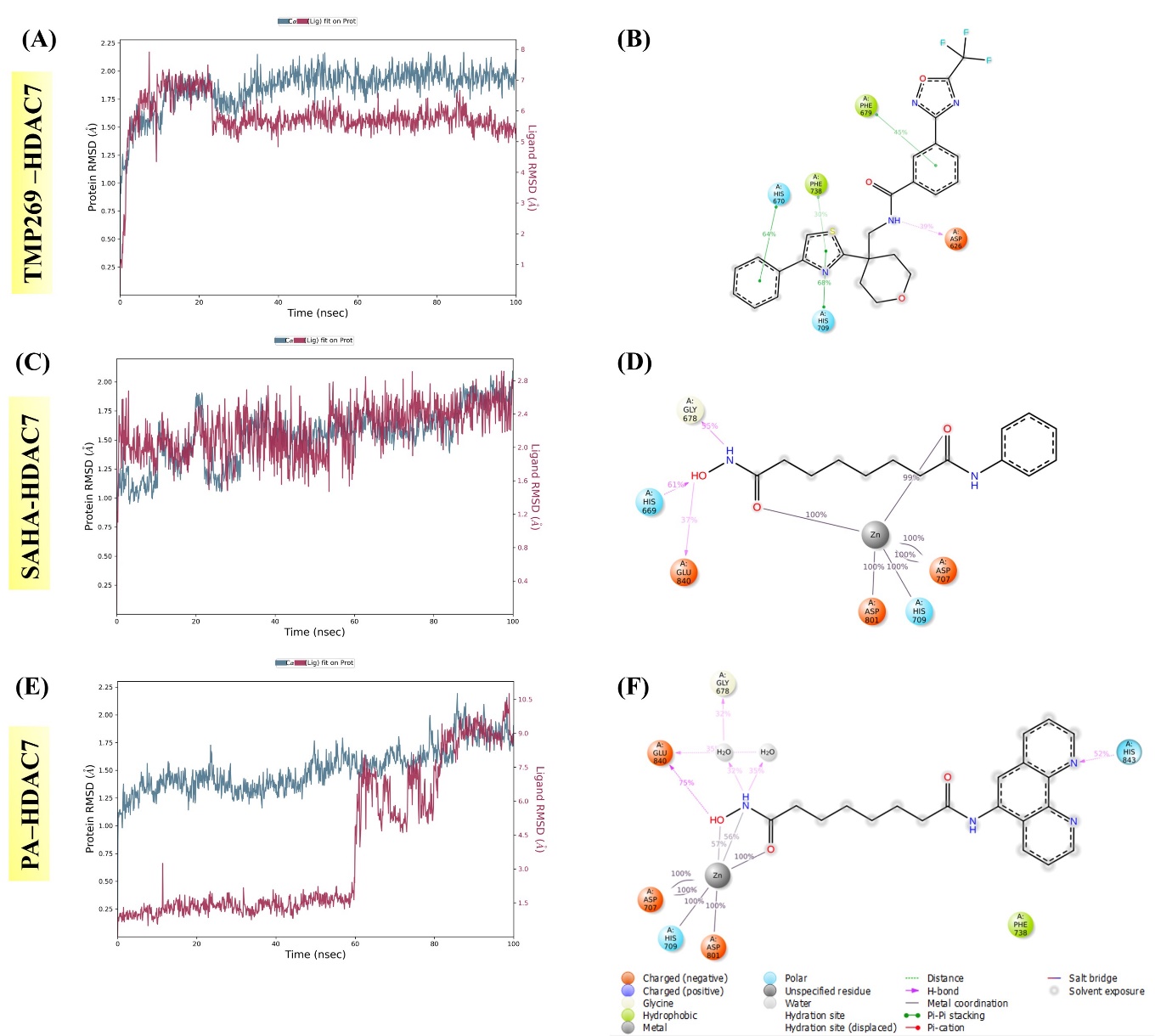


**Fig. S1** RMSD plot and 2D interaction of the HDAC7-ligand complex throughout the trajectory **(A) and (B)** TMP269-HDAC7, **(C) and (D)** SAHA-HDAC7, **(E) and (F)** PA-HDAC7.


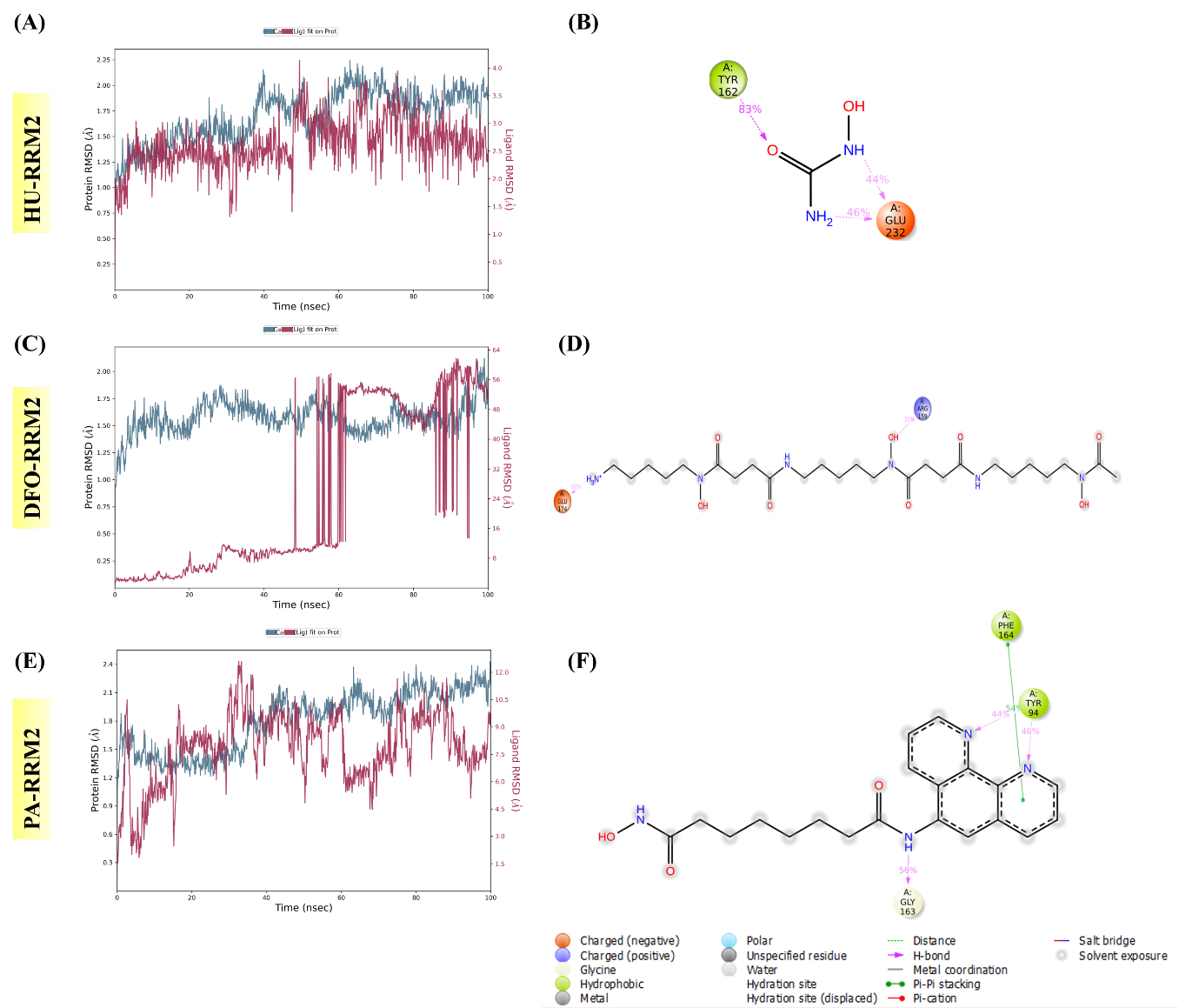


**Fig. S2** RMSD plot and 2D interaction of the RRM2-ligand complex throughout the trajectory **(A) and (B)** HU-RRM2, **(C) and (D)** DFO-RRM2, **(E) and (F)** PA-RRM2.

**Table S2:** Summary of the ligand-protein interactions in MD simulation.

| **Ligand-protein complex** | **H-bond** | **Pi-Pi interaction** | **Hydrophobic interaction** | **Polar interaction** | **Metal interaction** | **Other interaction** |
| --- | --- | --- | --- | --- | --- | --- |
| TMP269-HDAC7 | Asp626 | His670, Phe679, His709, Phe738 | Phe679, Phe738 | His670, His709 | - | Positive: Asp626 |
| HU-RRM2 | Tyr162, Glu232 | - | - | - | - | Positive: Glu232 |
| SAHA-HDAC7 | His669, Gly678, Glu840 | - | - | His669 | Zn | Positive: Asp707, Asp 801, Glu840 |
| DFO-RRM2 | Arg159, Glu174 | - | - | - | - | Positive: Glu174;  Negative: Arg159 |
| PA-HDAC7 | Glu840 | - | Phe738 | His708 | Zn | Positive: Asp707, Asp 801, Glu840 |
| PA-RRM2 | Tyr94, Gly163 | Phe164 | - | - | - | - |

*3.4.3 ADME prediction*

**Table S3:** *In silico* ADME profile of the compound.

| **Compound** | **Molecular weight**  **(g/ mol)** | **Number of**  **H bond donor** | **Number of**  **H bond acceptor** | **Log P (o/w)** | **Violation of Lipinski’s rule** |
| --- | --- | --- | --- | --- | --- |
| PA | 366.41 | 3 | 5 | 1.56 | 0 |
| 1,10-Phenanthroline | 180.21 | 0 | 2 | 1.65 | 0 |
| Hydroxyurea | 76.05 | 3 | 2 | -0.16 | 0 |
| SAHA | 264.32 | 3 | 3 | 1.84 | 0 |
| DFO | 560.68 | 6 | 9 | 4.33 | 1 |

**List of abbreviations**

| ATCC | American Type Culture Collection |
| --- | --- |
| CO_2_ | Carbon dioxide |
| CST | Cell signaling technology |
| DAPI | 4′,6-diamidino-2-phenylindole |
| DCFH-DA | Dichloro-dihydro-fluorescein diacetate |
| DFO | Deferoxamine |
| DFT | Density functional theory |
| DMEM | Dulbecco's Modified Eagle Medium |
| DMSO | Dimethyl sulfoxide |
| dNTP | Deoxynucleotide triphosphates |
| Et_3_N | Triethylamine |
| EtOAc | Ethyl acetate |
| FBS | Fetal Bovine Serum |
| FTIR | Fourier Transform Infrared Spectroscopy |
| GIT | Gastro-intestinal tract |
| RR | Ribonucleotide reductase |
| HAT | Histone acetyltransferase |
| HDAC | Histone deacetylase |
| HOMO | Highest occupied molecular orbital |
| HPLC | High-performance liquid chromatography |
| HU | Hydroxyurea |
| KOH | Potassium hydroxide |
| LUMO | Least unoccupied molecular orbital |
| MD | Molecular dynamic simulation |
| MeOH | Methanol |
| MOPS | 3-(N-morpholino)propanesulfonic acid |
| MP | Melting point |
| MTT | 3-[4,5-dimethylthiazol-2-yl]-2,5 diphenyl tetrazolium bromide |
| NH_2_OH.HCl | Hydroxylamine hydrochloride |
| NMR | Nuclear Magnetic Resonance |
| PDB | Protein data bank |
| RIPA | Radioimmunoprecipitation assay buffer |
| ROS | Reactive oxygen species |
| RRM2 | Ribonucleotide reductase regulatory subunit M2 |
| SAHA | suberoylanilide hydroxamic acid |
| SDS-PAGE | Sodium dodecyl-sulfate polyacrylamide gel electrophoresis |
| TDDFT | Time-dependent density functional theory |
| THF | Tetrahydrofuran |
| TLC | Thin layer chromatography |
| VC | DMSO (0.1%) |
| XP | Extra precision |
