# Supplementary material for: Evaluation of 1,10-phenanthroline-based hydroxamate derivative as dual histone deacetylases/ribonucleotide reductase inhibitor with antitumor activities
Source: Daru. 2024 Apr 29;32(1):263–78. doi: 10.1007/s40199-024-00514-1 (PMC11087398; doi:10.1007/s40199-024-00514-1)
Supplement: Supplementary file 2 — Supplementary file2 (DOCX 759 KB) [file 40199_2024_514_MOESM2_ESM.docx]

**Supplementary file-2**

***Article title:***

Evaluation of 1,10-Phenanthroline-Based Hydroxamate Derivative as Dual Histone Deacetylases/ Ribonucleotide Reductase Inhibitor with Antitumor Activities

***Journal name:***

DARU Journal of Pharmaceutical Sciences

***Author names:***

Manasa Gangadhar Shetty^1^, Padmini Pai^1#^, Bipasa Dey^1#^ Kapaettu Satyamoorthy^2^, Suranjan Shil^3^, Usha Yogendra Nayak^4^, Aswini T^4^, Babitha Kampa Sundara^1*^.

***Affiliation:***

^1^Department of Biophysics, Manipal School of Life Sciences, Manipal Academy of Higher Education, Manipal - 576104, Karnataka, India

^2^Shri Dharmasthala Manjunatheshwara (SDM) University, Manjushree Nagar, Sattur, Dharwad - 580009, Karnataka, India

^3^Department of Chemistry, Manipal Centre for Natural Sciences (Centre of Excellence), Manipal Academy of Higher Education, Manipal - 576104, Karnataka, India

^4^Department of Pharmaceutics, Manipal College of Pharmaceutical Sciences, Manipal Academy of Higher Education, Manipal, Karnataka 576104, India

***e-mail address of the corresponding author.***

*babitha.ks@manipal.edu

MS spectrum of PA


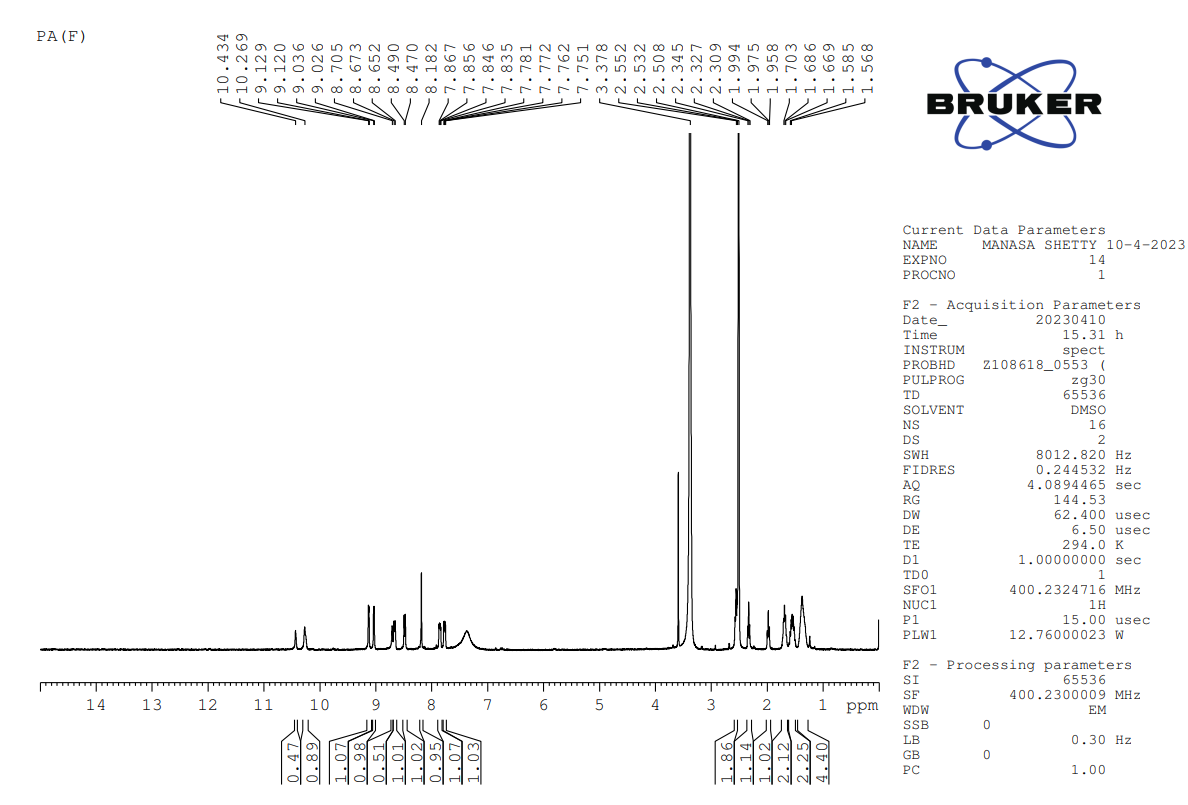


^1^HNMR spectrum of PA


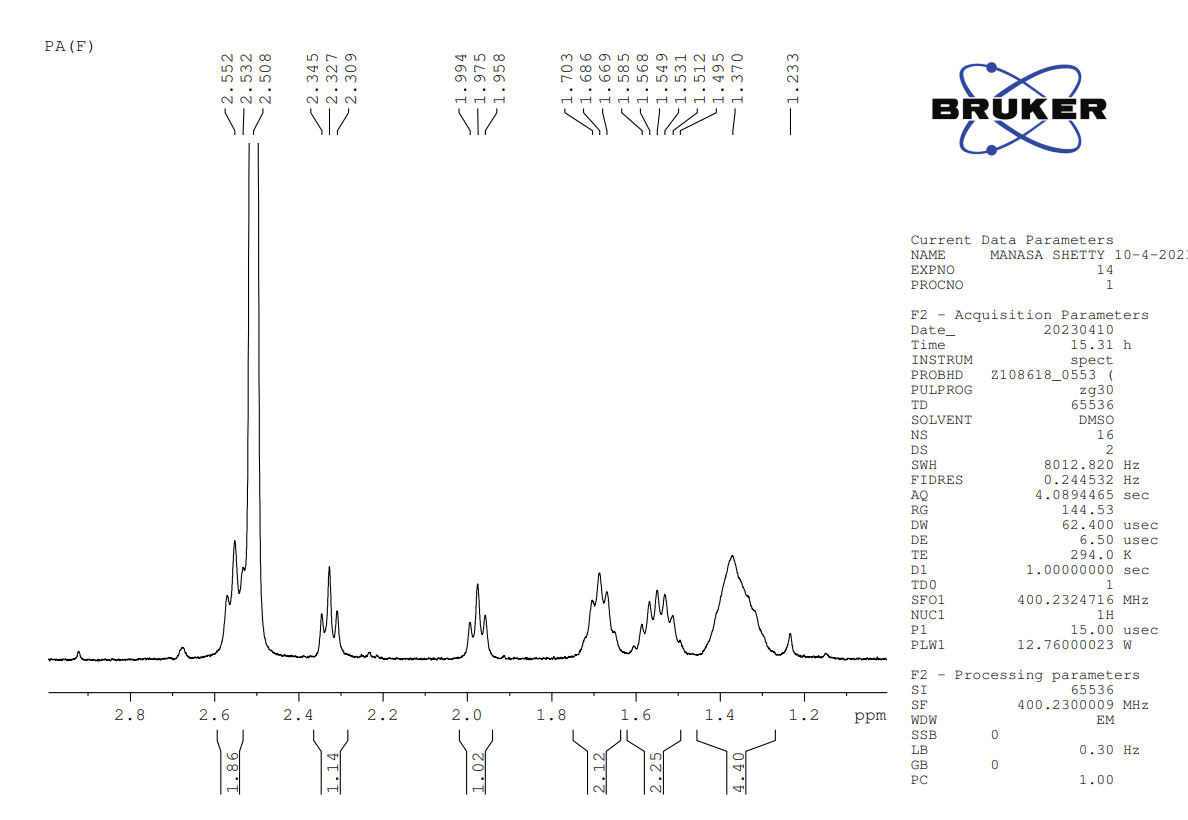


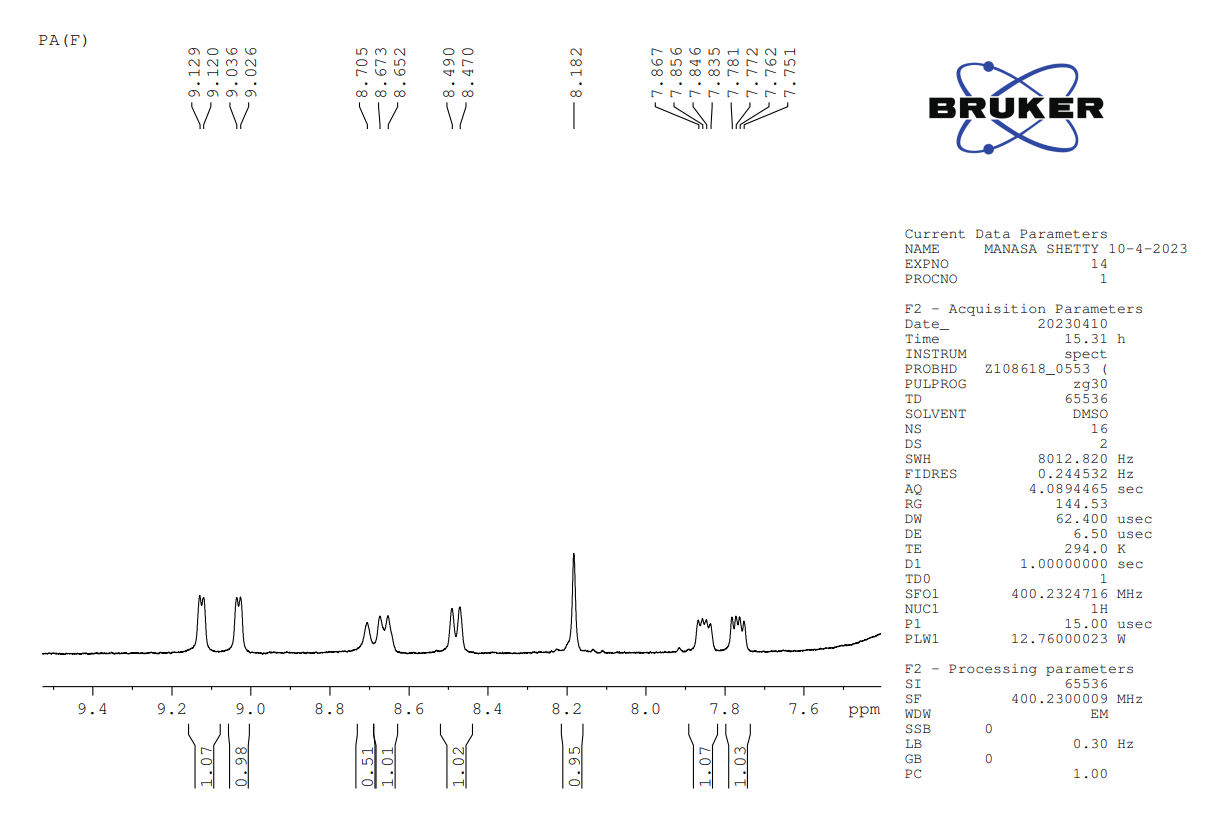


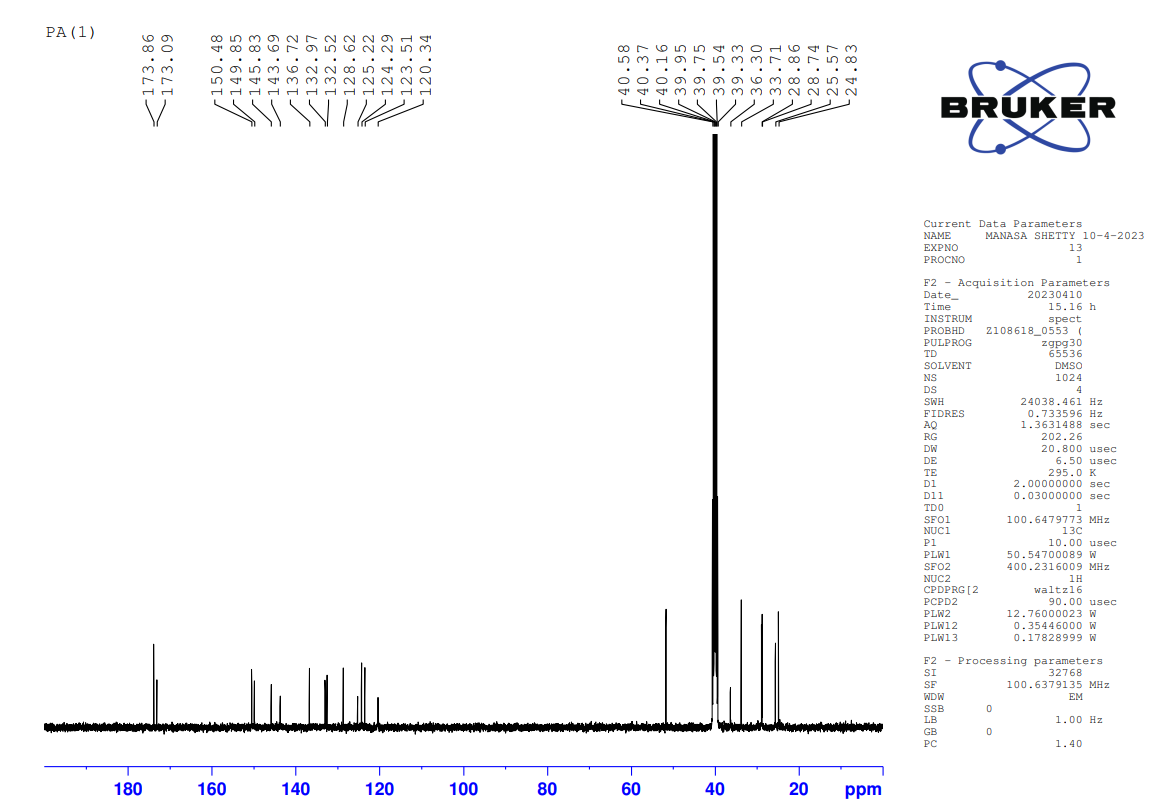


^13^C NMR OF PA


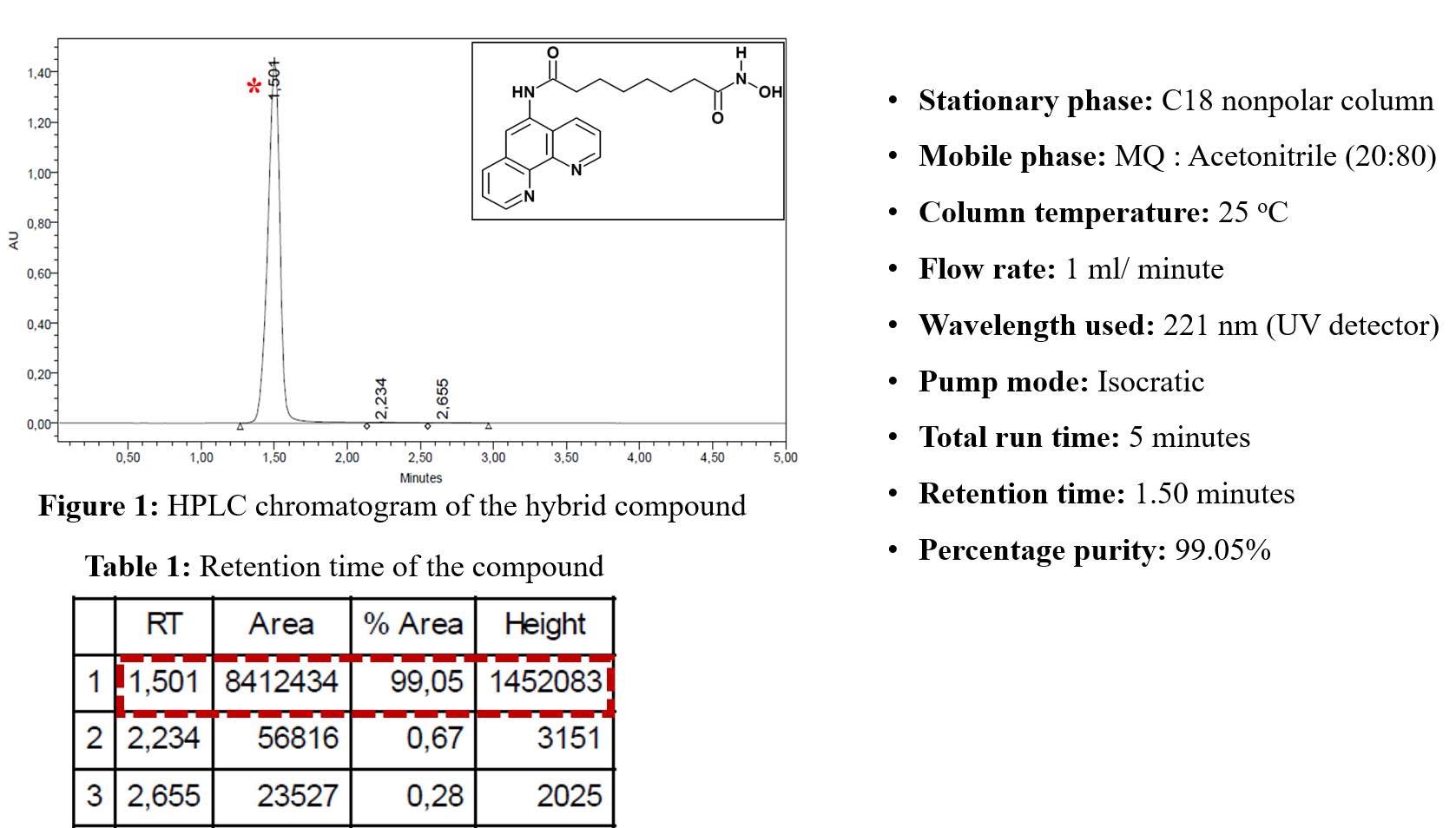


HPLC of PA
